# Multi-tissue and multi-scale approach for nuclei segmentation in H&E stained images

**DOI:** 10.1186/s12938-018-0518-0

**Published:** 2018-06-20

**Authors:** Massimo Salvi, Filippo Molinari

**Affiliations:** Biolab, Department of Electronics and Telecomunications, Politecnico di Torino, 10129 Turin, Italy

**Keywords:** Nuclei segmentation, Adaptive thresholding, Cellular imaging, Computer-aided image analysis

## Abstract

**Background:**

Accurate nuclei detection and segmentation in histological images is essential for many clinical purposes. While manual annotations are time-consuming and operator-dependent, full automated segmentation remains a challenging task due to the high variability of cells intensity, size and morphology. Most of the proposed algorithms for the automated segmentation of nuclei were designed for specific organ or tissues.

**Results:**

The aim of this study was to develop and validate a fully multiscale method, named MANA (Multiscale Adaptive Nuclei Analysis), for nuclei segmentation in different tissues and magnifications. MANA was tested on a dataset of H&E stained tissue images with more than 59,000 annotated nuclei, taken from six organs (colon, liver, bone, prostate, adrenal gland and thyroid) and three magnifications (10×, 20×, 40×). Automatic results were compared with manual segmentations and three open-source software designed for nuclei detection. For each organ, MANA obtained always an F1-score higher than 0.91, with an average F1 of 0.9305 ± 0.0161. The average computational time was about 20 s independently of the number of nuclei to be detected (anyway, higher than 1000), indicating the efficiency of the proposed technique.

**Conclusion:**

To the best of our knowledge, MANA is the first fully automated multi-scale and multi-tissue algorithm for nuclei detection. Overall, the robustness and versatility of MANA allowed to achieve, on different organs and magnifications, performances in line or better than those of state-of-art algorithms optimized for single tissues.

## Background

The evaluation of cell nuclei plays a crucial role in histopathological images analysis. In fact, parameters such as cell size, shape and spatial distribution are generally used by pathologists for cancer detection and reporting [1]. In routine histology, the most widely used staining method to visualize tissues is the use of hematoxylin and eosin (H&E), which allow to distinguish cell nuclei (bluish color—hematoxylin) from cytoplasm (pinkish color—eosin) [2]. Cell nuclei counting is time-consuming and prone to inter- and intra-observer variability, which results in a limited reliability. Manual delineation of nuclei is an even more cumbersome operation, which is never performed in routine, but which would be required to precisely assess nuclei size and morphology. The architectural arrangement of nuclear structures on histology is highly relevant in the context of disease (i.e., cancer grading) [3]. Cancer grade is a key feature used to predict patient prognosis and in prescribing a treatment [1]. Since most of the current pathology diagnosis processes are based on the subjective opinion of pathologists, solutions for the quantitative assessment of histological images would have scope of application.

With the recent advances of techniques in digitalized scanning, tissue histopathology slides can be stored in the form of digital images [4]. In the last years, many efforts have been devoted to developing automatic nuclear segmentation techniques with the aim to improve the efficiency and the accuracy in histopathological image analysis.

Most current nuclei detection approaches on H&E stained images are based on color information [5, 6]. Using these techniques, a detection accuracy over 85% can be achieved [7]. Since these approaches are dependent on either color and intensity-related attributes, none of these works have been tested on multi-tissue data or in pathological conditions, where nuclei may exhibit irregular shapes and different intensities.

Several methods have been proposed to perform cell segmentation using gradients [8] and morphological operations [9]. Nevertheless, methods using a prior knowledge of nuclei shape are prone to fail because of the variation of tissue preparation procedures (sectioning and staining). Furthermore, the existence of touching nuclei makes their separation quite hard for automated segmentation methods [4].

In the last few year, deep neural networks drove advances in image recognition and they achieved state-of-art performance in many segmentation tasks of medical imaging [10, 11]. Above all, convolutional neural networks (CNNs) have shown promising results in nuclei segmentation for different tissues [12]. These techniques estimate a probability map of the nuclear regions based on the learned nuclear appearances. In this way, CNNs can generalize across various nuclear color variations. Recently, a detection accuracy of 80% was obtained for seven organs [12]. However, CNNs need a wide annotated training set of images to obtain adequate performance and the network architecture must be changed in case of variation in the magnification. This is because CNNs fail to generalize if the nuclei, in addition to changing color, also change size. For this reason, deep neural networks are not suitable for multiscale approaches.

To the best of our knowledge, no multi-tissue and multi-scale solution has been proposed so far. In this paper, we present the MANA (Multiscale Adaptive Nuclei Analysis) algorithm, a multi-tissue and multi-scale method for cell detection in histological images. The proposed technique takes an H&E staining image as input and it shows the nuclei boundaries found within the image.

## Methods

The MANA algorithm was designed to automatically detect nuclei in H&E staining images. The algorithm was developed using MATLAB (MathWorks, Natick, MA, USA) environment. Three main steps composed the processing: object-based thresholding, area-based correction and nuclei separation. In the following sections, a detailed description of the algorithm is provided.

### Object-based detection

This step represents our technical innovation to achieve a first object-based detection for nuclei segmentation. The RGB image of a histological specimen is first converted into grayscale by eliminating the hue and saturation information while retaining the luminance. Then, its histogram is calculated and the progressive weighted mean (*PWM*_*CURVE*_) of the grayscale histogram is computed.

Let’s consider a grayscale image with pixel intensities expressed by integer numbers between 0 and N. The histogram is then a distribution with N + 1 classes and it graphically displays the frequency (how many times) each gray level occurs. Considering a generic class P of the histogram (0 ≤ P ≤ N), the value of *PWM*_*CURVE*_ for that class is defined as follows:

$$PWM_{CURVE} \, = \, \frac{{\mathop \sum \nolimits_{i\, = \,0}^{P} w_{i} x_{i} }}{{\mathop \sum \nolimits_{i\, = \,0}^{P} w_{i} }}$$

where *w*_*i*_ is the histogram count for the *i*th class and *x*_*i*_ is the respective bin location. The *PWM*_*CURVE*_ is evaluated for each class of the histogram as the weighted mean of all the grayscale histogram values up to that class. The trend of *PWM*_*CURVE*_ depends on the histogram shape so relevant characteristics on the color distribution of the image can be extracted using this function. In particular, if there are significant color variations from a certain point on the histogram with respect to the distribution that precedes it, here we can expect to see a change of concavity in the *PWM*_*CURVE*_. Inflection points of *PWM*_*CURVE*_ may be potential threshold values for performing nuclei segmentation as they represent local stability points of the grayscale histogram.

Conceptually, *PWM*_*CURVE*_ is therefore an alternative representation of the color distribution that makes it easier to apply object-based thresholds. For this reason, *PWM*_*CURVE*_ can be used to automatically spot nuclei inside image. Nuclei are defined as objects with an intensity lower than a threshold.

First of all, the *PWM*_*CURVE*_ is fitted with a 15th order polynomial function with the aim to estimate its inflection points (*candidate thresholds*). Then, the grayscale image is segmented using all the candidate thresholds and the median area of objects found is evaluated for all thresholds. Among all the candidate thresholds, the algorithm defined as the *initial threshold* the one that had the objects with the highest median area.

**Fig. 1:**
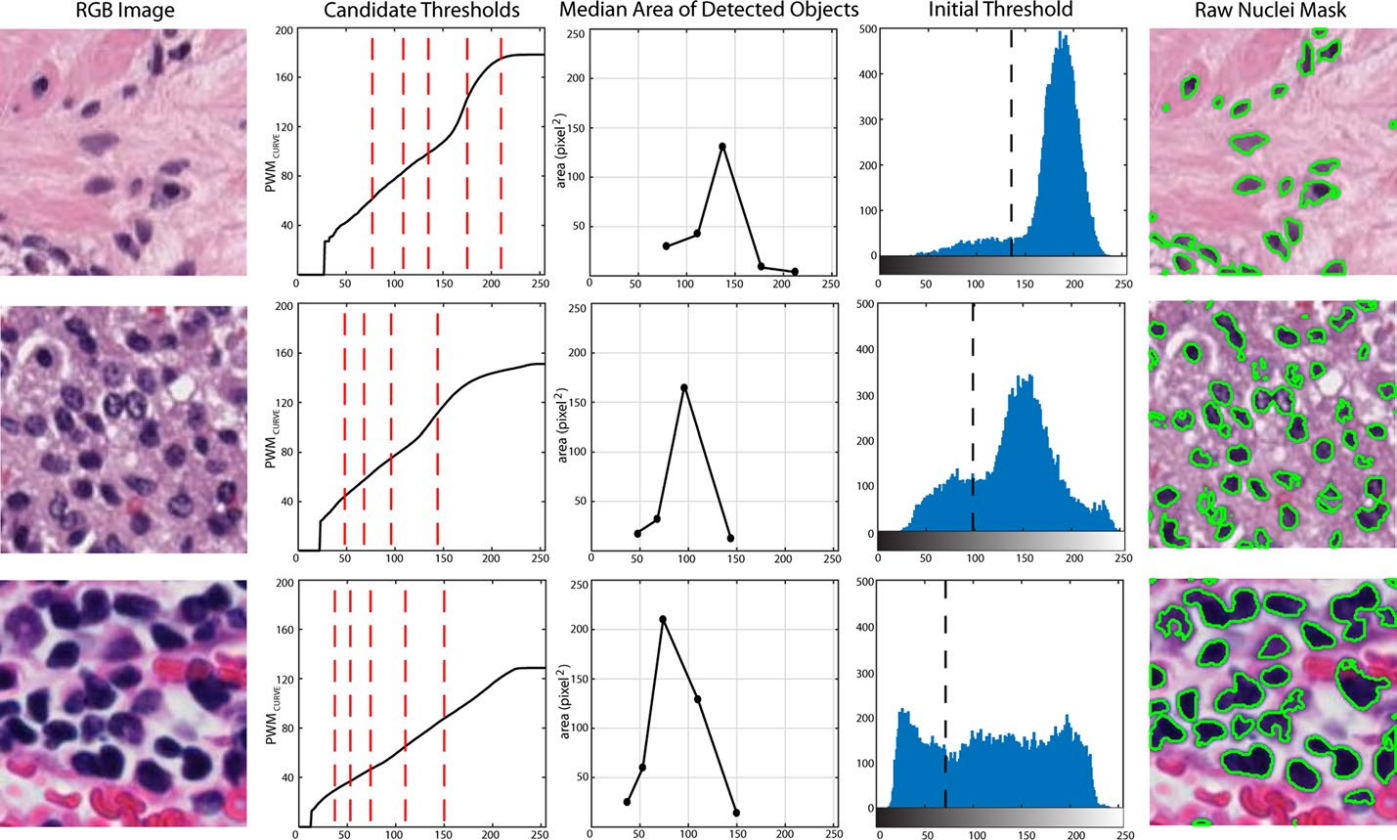
Processing for obtaining the initial threshold for different tissues (rows), showing images with a high variation of cells number, size and color. Starting from the RGB image, the PWM_CURVE_ is estimated from its grayscale histogram. Then, candidate thresholds are evaluated as inflection points of the curve (red dotted lines). The median area of detected objects using candidate thresholds is calculated and the initial threshold is determined as the one with the highest median area. In the last column, the application of the initial threshold on the RGB image is shown

The processing for obtaining the initial threshold is illustrated in Fig. 1, where three sub-images from different tissues are used as examples. Figure 1 also shows the robustness of the proposed method, where the optimal threshold was chosen, regardless of the histogram shapes or cells’ appearance.

By summarizing, being this method an object-based thresholding, it is robust to different tissue types, image magnification, and staining.

### Area-based correction

This step is needed in order to correct oversegmentation from previous step because the object-based detection may lead to small or too large structures. Too small structures may be oversegmented or wrong objects, whereas too large areas may consist of a fusion of different nuclei. To lessen the oversegmentation and to optimize the nuclei detection, the mean area of segmented objects (*mean total*) is first evaluated. Then, areas are labelled as: ‘small’, ‘normal’, or ‘big’. ‘Small’ objects are structures smaller than 25% of mean area, whereas ‘big’ objects are structures greater than 5 times the mean area. The remaining objects are considered as ‘normal’.

**Fig. 2:**
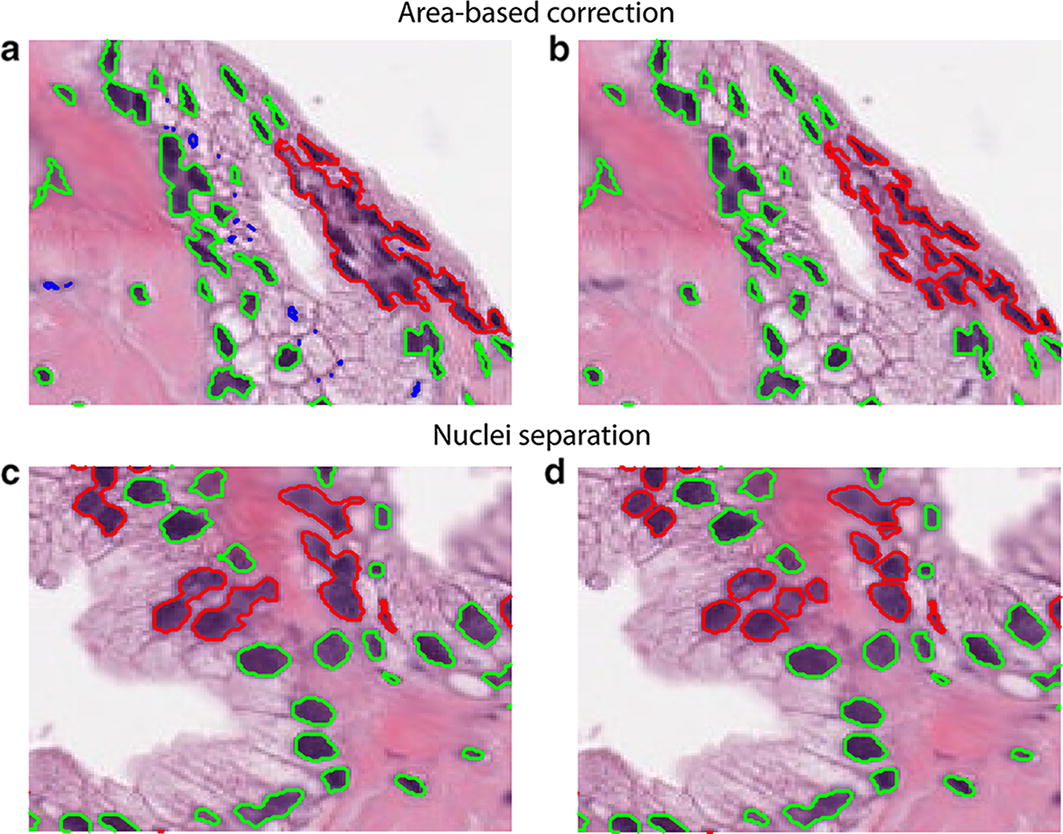
MANA processing steps. Left column reports the input image and the right one represents the output of the corresponding step. First row is relative to the areas evaluation (**a**, **b**) where small objects (blue) are deleted and big structures (red) are partially divided. Second row shows the nuclei separation (**c**, **d**) in which a marker-based watershed is applied on objects with small (red) and high solidity (green)

‘Small’ objects are deleted because they are too little to be potentially considered as nuclei. ‘Big’ objects should be split, in case they were nuclei agglomerates. Separation is obtained by iteratively decreasing the initial threshold for these structures until they are classified as ‘normal’ (area less than 5 times the mean total). Figure 2a sketches the effect of this procedure. Using these criteria, the initial threshold found in the previous section is locally modified in order to identify the highest number of nuclei within the histological image.

### Nuclei separation

The goal of this step is to further separate remaining fused nuclei. In literature, the watershed transform was successfully used to isolate merged nuclei [13]. The MANA algorithm implements a variant of the classical watershed transform called marker-based watershed [14]. In this technique, seeds close to nuclear centers (marker) are used as starting points for watershed transform. To identify nuclear seed, MANA performs the distance transform of the nuclei binary mask and calculates the local maxima using the extended-maxima transform [15]. This transform estimates the regional maxima by searching in N-connected neighborhoods. The neighborhood size determines the sensitivity of the maxima-extended transform in the detection of nuclear seeds.

Additionally, the solidity of all objects is also evaluated. Solidity of a region is defined as the ratio between its actual area and its convex area. Since it is expected that nuclei are convex objects, a segmented region containing an actual nucleus should have a solidity approximately equal to 1. Hence, solidity can be used as a discriminant feature for varying the neighborhood size of the maxima-extended transform and then the sensitivity of the marker-based watershed. The MANA algorithm applied a low-sensitive watershed for high solidity shapes while sensitivity is increased for low solidity objects. In Fig. 2b is shown the application of a marker-based watershed sensitive to shapes solidity.

Finally, the mean area of the objects obtained after watershed is evaluated and items smaller than 25% of mean area are erased by the algorithm.

### Performance measures

Automatic results provided by MANA were compared with manual segmentations. True positive (TP) represents the number of manual cells identified by the algorithm, false negative (FN) denotes all nuclei not found by the automatic method and false positive (FP) represents all cells obtained by MANA without a corresponding manual nucleus. The performance of nuclear detection was evaluated by calculating the recall, precision and F1-score, which are defined as follows:

$$recall \, = \, \frac{TP}{TP\, + \,FN}$$

$$precision\, = \, \frac{TP}{TP\, + \,FP}$$

$$F1\, = \,\frac{{2 \, \times \, \left( {recall \, \times \,precision} \right)}}{{\left( {recall\, + \,precision} \right)}}.$$

**Fig. 3:**
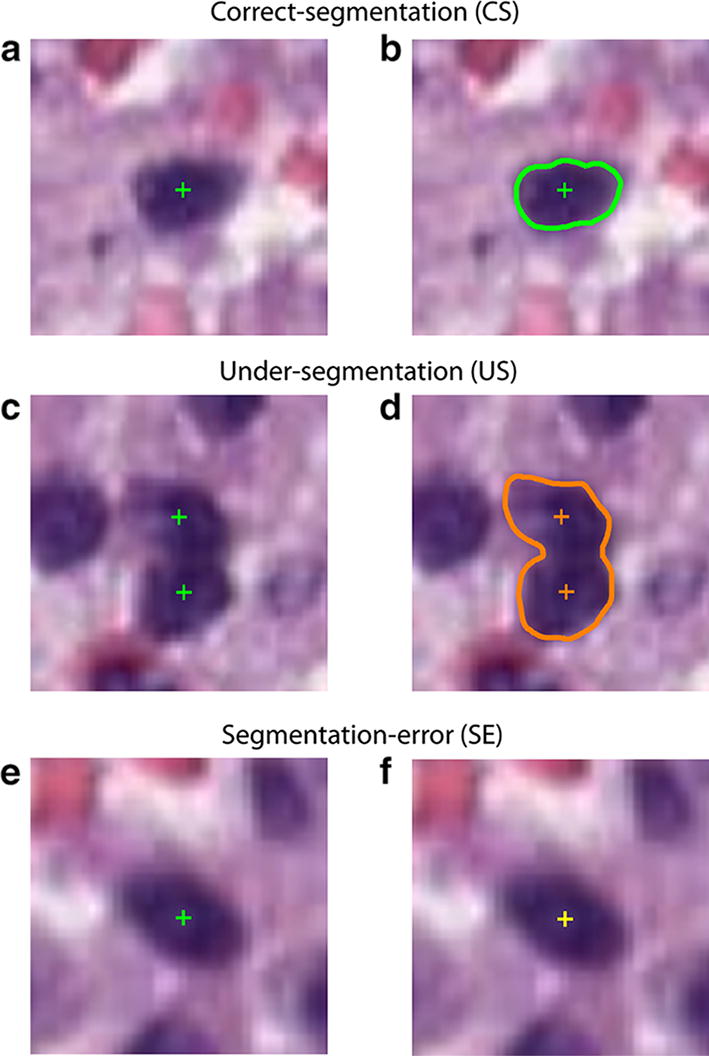
Pixel-level performance. Left column illustrates the manual annotations and the right one shows the corresponding automatic results. An example of (**a**, **b**) correct-segmentation (green), (**c**, **d**) under-segmentation (orange), (**e**, **f**) segmentation-error (yellow)

Recall assesses the missed detection of ground truth objects (manual nuclei) while precision evaluates the false detection of ghost objects. F1-score is defined as the harmonic mean of recall and precision. F1-score is a common used object detection metric [16], but it penalizes only object-level errors [12]. In fact, F1-score does not take pixel-level errors into account (i.e. under-segmentation of correctly detected objects). Let N_CS_, N_US_ and N_SE_ represent the numbers of correct-segmentation (CS), under-segmentation (US) and segmentation-error (SE). The pixel-level performance is evaluated using the CS, US and SE rates [17], which are defined as follows:

$$CS \, = \, \frac{{N_{CS} }}{{N_{GT} }}\, \times \, 100\%$$

$$US\, = \, \frac{{N_{US} }}{{N_{GT} }}\, \times \,100\%$$

$$SE\, = \, \frac{{N_{SE} }}{{N_{GT} }}\, \times \, 100\%$$

where N_GT_ (ground truth) represents the number of nuclei manually identified. The US rate indicates the failure to split nuclear regions in the correct number of nuclei while SE rate reveals the missed detection of cells. An example of CS, US and SE cells is provided in Fig. 3.

## Results

Our dataset consisted of H&E stained images taken from six different organ tissues. The six organs were: colon, liver, bone, prostate, adrenal gland and thyroid. In addition, images were acquired with three magnifications (10×, 20×, 40×) to test the multiscale approach of MANA algorithm. One expert pathologist (more than 10 years of experience) manually marked the nuclei centers in each image, for a total of 59,123 cells. The images were collected and digitalized at the Molinette Città della Salute University hospital (Torino, Italy) and all patients signed an informed consent. The overall dataset composition is shown in Table 1.

**Table 1:** Dataset composition

Tissue	Magnifications	#Nuclei
Colon	20×	9166
Liver	10×, 20×	5051
Bone	40×	6889
Prostate	20×	5995
Adrenal gland	10×, 20×	12,972
Thyroid	10×, 20×	19,050
Total	10×, 20×, 40×	59,123

**Fig. 4:**
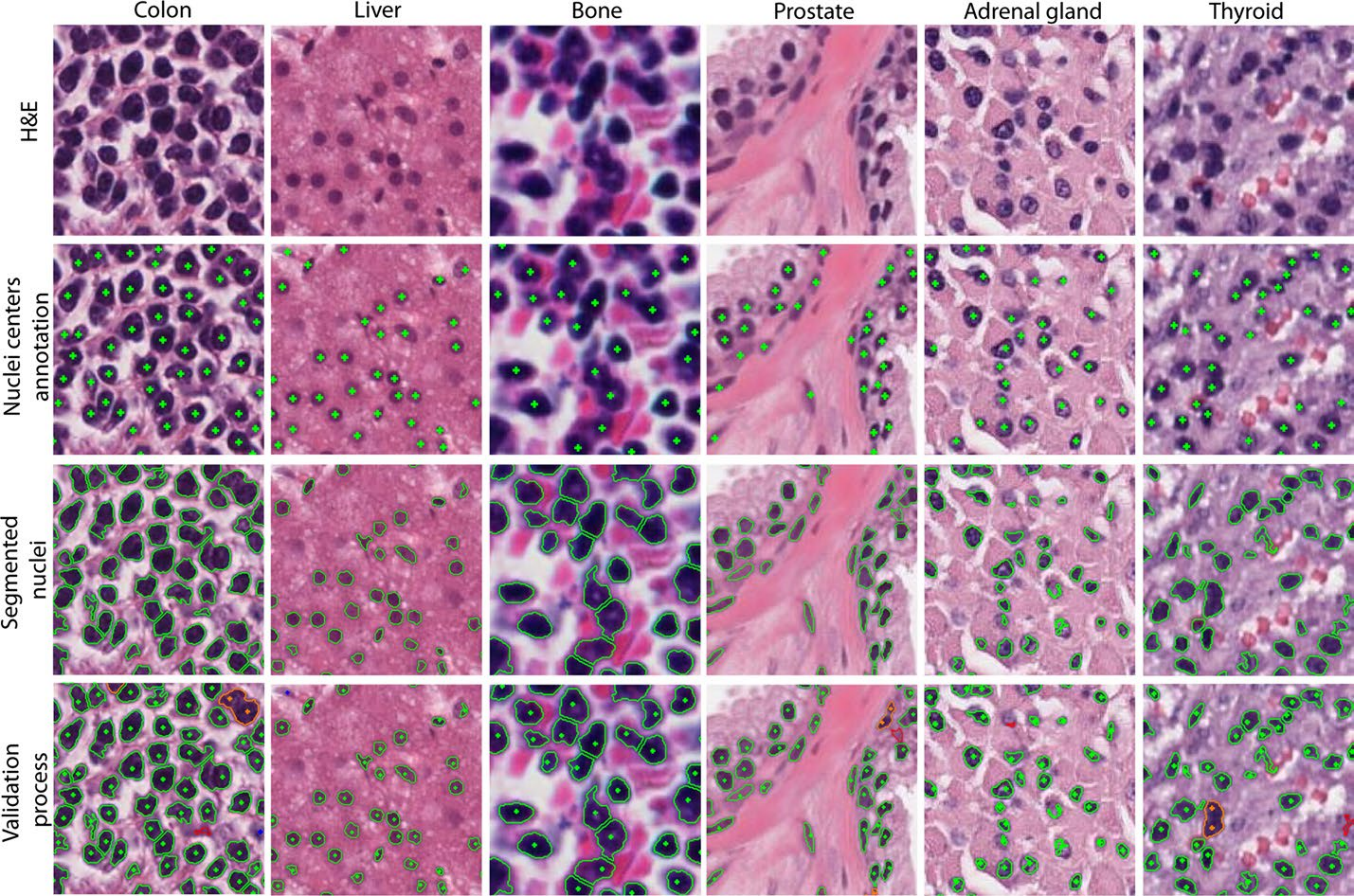
Examples of sub-images taken from different tissues (columns), showing challenging cases based on variation in nuclear appearance, crowding and dimension. Manual annotation, automatic segmentation and validation process are shown in rows. In the last row, true positive cells are highlighted in green while false negative and false positive nuclei are shown in blue and red respectively. Finally, under-segmented nuclei are illustrated in orange

For each of the six organs analyzed, an example of the validation process is shown in Fig. 4.

### Comparison with manual operator

The object-level (recall, precision and F1-score) and pixel-level (CS, US, SE rates) performances of MANA algorithm are summarized in Table 2. The processing was performed on a workstation with a 2.6 GHz quad-core CPU and 16-GB of RAM.

**Table 2:** Performances of the proposed method

Organ	Computational time (s)	Object-level performance			Pixel-level performance		
		Recall	Precision	F1-score	CS (%)	US (%)	SE (%)
Colon	22.89 ± 2.15	0.9505 ± 0.0121	0.9048 ± 0.0114	0.9270 ± 0.0086	86.78 ± 1.97	8.69 ± 2.00	4.53 ± 1.14
Liver	11.32 ± 1.25	0.9249 ± 0.0267	0.9547 ± 0.0114	0.9392 ± 0.0101	87.17 ± 4.55	5.81 ± 2.42	7.02 ± 2.37
Bone	13.10 ± 1.13	0.9486 ± 0.0290	0.9362 ± 0.0203	0.9417 ± 0.0077	74.15 ± 4.17	21.35 ± 4.30	4.07 ± 2.49
Prostate	12.28 ± 1.31	0.9533 ± 0.0127	0.9404 ± 0.0147	0.9467 ± 0.0106	77.47 ± 7.72	18.79 ± 7.31	3.74 ± 0.88
Adrenal gland	18.02 ± 1.26	0.9126 ± 0.0300	0.9239 ± 0.0312	0.9174 ± 0.0129	84.60 ± 4.44	7.33 ± 2.55	8.06 ± 2.62
Thyroid	23.71 ± 5.94	0.9335 ± 0.0296	0.8914 ± 0.0221	0.9112 ± 0.0038	81.62 ± 6.18	12.61 ± 5.39	5.77 ± 2.60
Overall	16.89 ± 5.72	0.9372 ± 0.0288	0.9253 ± 0.0293	0.9305 ± 0.0161	81.97 ± 7.05	12.43 ± 7.32	5.53 ± 2.66

Data are reported as mean ± standard deviation

The algorithm can be considered very performing in object detection, being the average F1-score equal to 0.9305 on 30 images. For all tissues, precision and recall presented similar values so the accuracy of the proposed method was demonstrated (Table 2).

A CS rate of 81.97% coupled to a SE rate of 5.53% was also obtained. Moreover, the US rate was small where nuclei had crisp contours (5.81%) while it increased in organs with a high percentage of touching nuclei (21.35%).

Finally, the computational time is slightly dependent on image resolution, ranging between 11.3 and 23.7 s (average ± SD: 16.89 ± 5.72 s).

### Benchmarking with open-source software

**Fig. 5:**
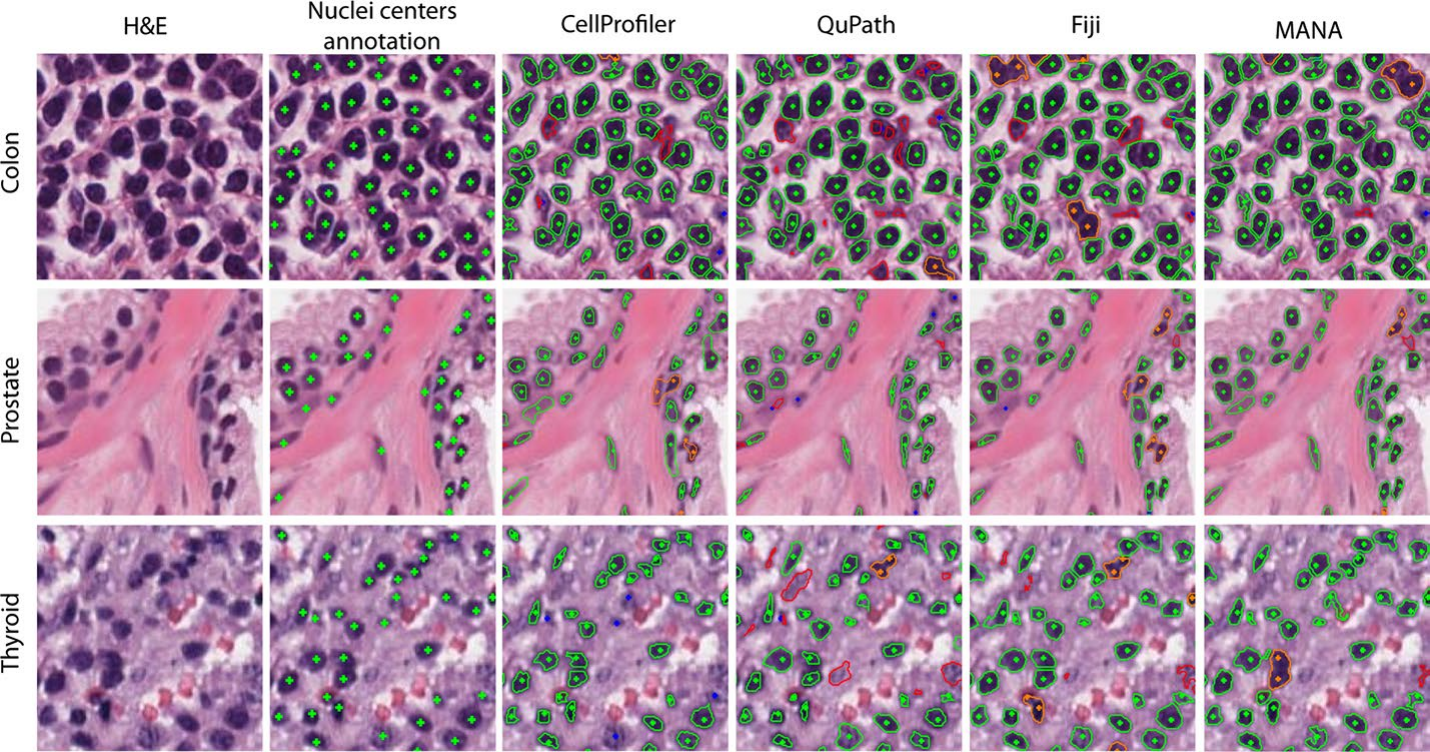
Comparison between three open-source software and the proposed method in the nuclei detection. Sub-images from different tissues are shown in rows while cell segmentation results are illustrated in columns

The results obtained by the proposed algorithm were also compared with three open-source software (CellProfiler, QuPath and Fiji) used in the analysis of histological images [18]. CellProfiler [19] allows to create pipelines for the processing of biomedical images. The software is composed of a series of image-processing modules that allow the user to perform an automatic analysis of the histological images. QuPath [20] is a new bioimage analysis software designed to provide an open-source solution for digital pathology and whole slide image analysis. This software allows to perform several automatic analyses of histological images, including nuclei detection. Fiji [21] is a Java-based software that has a watershed transform-based nuclear segmentation plugin available. For this software, a semi-automatic pipeline was implemented, consisted of: (i) conversion of H&E image into grayscale, (ii) manual intensity thresholding and (iii) automatic cells separation. The comparison in the nuclei detection of CellProfiler, QuPath, Fiji and MANA is provided in Fig. 5. The performances of the three open-source software are also reported in Table 3.

**Table 3:** Performances of three open-source software for nuclei detection (CellProfiler, QuPath, Fiji)

Method	Computational Time (s)	Object-level performance			Pixel-level performance		
		Recall	Precision	F1-score	CS (%)	US (%)	SE (%)
CellProfiler (automatic)	21.13 ± 3.78	0.6866 ± 0.2421	0.8274 ± 0.0820	0.7154 ± 0.2030	65.47 ± 23.23	4.79 ± 3.21	29.73 ± 23.44
QuPath (automatic)	11.37 ± 1.96	0.9248 ± 0.0552	0.7120 ± 0.0916	0.8004 ± 0.0638	74.24 ± 10.41	9.29 ± 7.70	6.47 ± 3.23
Fiji (semi-automatic)	252.73 ± 76.11	0.9462 ± 0.0386	0.8658 ± 0.0424	0.9030 ± 0.0248	82.59 ± 7.05	12.69 ± 5.88	4.65 ± 3.30
MANA (proposed)	*16.89 ± 5.72*	*0.9372 ± 0.0288*	*0.9253 ± 0.0293*	*0.9305 ± 0.0161*	*81.97 ± 7.05*	*12.43 ± 7.32*	*5.53 ± 2.66*

Data are reported as mean ± standard deviation

As can be seen from Table 3, the CellProfiler segmentation is characterized by a low recall. Several nuclei are not identified by the software and this generate a high number of false negative cells. The average F1-score (0.7154) was lower than the proposed one for more than 20%. This software had also a poor pixel-level performance, with a low number of correct-segmentation (65.45%) and a high number of segmentation-errors (SE equal to 29.73%).

QuPath proved to be an efficient tool for nuclei detection, with fast mean computational time (11.37 s) and average recall of about 0.93. On the other hand, this method produced a lot of false-positive nuclei, causing a very low precision (0.7120). This low precision leaded to a lowering of the average F1-score (0.8004).

The average F1-score obtained with Fiji was slightly lower than those achieved with MANA (0.9030 vs 0.9305). In fact, Fiji processing is based on a single threshold while the proposed method can locally modify the threshold on the same image in order to identify the highest number of nuclei. Moreover, the average computational time in Fiji was 252.73 s, about 15 times higher than MANA algorithm.

## Discussion

In the present study, we proposed a fully automatic method for nuclei identification in histological images. The cell nuclei segmentation is crucially important and has a wide range of applications, such as cancer diagnosis [22], cancer grading [23] and quantification of molecular markers in healthy and pathological specimens [9]. The proposed method is able to recognize nuclei boundaries inside H&E images. The cells detection in histological images is a challenging task because of nuclei variability in shape, intensity and dimension. Our technique did not require any user interaction and it was capable of automatically detecting nuclei in different tissues and magnifications. We chose to analyze six of the most studied organs in the development of automatic nuclei segmentation [24, 25]. Nuclei centers were manually marked by one expert pathologist, for a total of 59,123 cells. It was not necessary to segment nuclei boundaries since the proposed algorithm does not require a training set as deep learning-based methods. For this reason, having a faster manual segmentation, the number of annotated nuclei was increased, creating a dataset that contains more than twice the number of marked nuclei compared to previous works [12, 22, 26].

The automatic method was validated using metrics that penalizes both detection and segmentation errors. The comparison between manual and automatic segmentation showed high performances of the proposed technique. For each organ, MANA algorithm obtained always an F1-score higher than 0.91, with an average F1 of 0.9305 ± 0.0161. In literature, the only multi-tissue nuclei segmentation system [12] had an average F1-score of 0.8267. Compared with this state-of-art method, our approach achieved a large margin with 10.38% improvement of the identification rate.

Object-level and pixel-level performances were also comparable to previous works on nuclei detection [24, 27, 28]. Overall, the robustness and versatility of MANA allowed to achieve, on several organs and magnifications, performances in line or better than those of state-of-art algorithms designed for single tissues [19, 20].

The proposed algorithm allowed also to obtain the highest average F1-score compared to other open-source software designed for nuclei detection. MANA had one of the lowest computational time and, respect to other automatic methods, it had the best pixel-level performances.

Thanks to the reliable nuclei detection provided by MANA, automated systems for tumor patterns recognition [29], histological lesions evaluation [25] and markers quantification [9] can be easily developed in a straightforward manner. In the future, a novel cells separation will be implemented to further increase the pixel-level performances of the proposed algorithm. Future studies are also required to test the accuracy of MANA algorithm for nuclei detection in other tissues.

## Conclusions

In this paper, an adaptive method for nuclei segmentation in H&E stained images is presented. To the best of our knowledge, MANA is the first fully automated multi-scale and multi-tissue algorithm for nuclei detection.

The algorithm was tested on different organs, in which nuclei had different intensities, shapes and dimensions. High segmentation performances were obtained for each image of the dataset. The observed robustness in nuclei detection provided by MANA was mainly due to the use of an adaptive thresholding and an optimized nuclei separation. The algorithm took around 20 s to perform segmentation in images with 1500 nuclei, indicating the efficiency of the proposed technique.

Being totally automated, this algorithm could be used in future studies as starting point to realize reliable systems for morphological tissue characterization and diagnosis. Our research group is currently working on a MANA-based algorithm for the automatic detection and quantification of tumor areas in different histological tissue.

## Abbreviations

MANA: Multiscale Adaptive Nuclei Analysis
H&E: hematoxylin and eosin
CNNs: convolutional neural networks
PWM_CURVE_: progressive weighted mean
TP: true positive
FN: false negative
FP: false positive
CS: correct-segmentation
US: under-segmentation
SE: segmentation-error
N_GT_: ground truth
